# Outlining eicosanoid biosynthesis in the crustacean *Daphnia*

**DOI:** 10.1186/1742-9994-5-11

**Published:** 2008-07-14

**Authors:** Lars-Henrik Heckmann, Richard M Sibly, Martijn JTN Timmermans, Amanda Callaghan

**Affiliations:** 1 University of Reading, School of Biological Sciences, Environmental Biology, PO Box 68, Reading, RG6 6BX, UK; 2 University of Aarhus, National Environmental Research Institute, Department of Terrestrial Ecology, Vejlsøvej 25, PO Box 314, DK-8600, Silkeborg, Denmark; 3 VU University Amsterdam, Institute of Ecological Science, Department of Animal Ecology, De Boelelaan 1085, 1081 HV, Amsterdam, The Netherlands

## Abstract

**Background:**

Eicosanoids are biologically active, oxygenated metabolites of three C20 polyunsaturated fatty acids. They act as signalling molecules within the autocrine or paracrine system in both vertebrates and invertebrates mainly functioning as important mediators in reproduction, the immune system and ion transport. The biosynthesis of eicosanoids has been intensively studied in mammals and it is known that they are synthesised from the fatty acid, arachidonic acid, through either the cyclooxygenase (COX) pathway; the lipoxygenase (LOX) pathway; or the cytochrome P450 epoxygenase pathway. However, little is still known about the synthesis and structure of the pathway in invertebrates.

**Results:**

Here, we show transcriptomic evidence from *Daphnia magna *(Crustacea: Branchiopoda) together with a bioinformatic analysis of the *D. pulex *genome providing insight on the role of eicosanoids in these crustaceans as well as outlining a putative pathway of eicosanoid biosynthesis. *Daphnia *appear only to have one copy of the gene encoding the key enzyme COX, and phylogenetic analysis reveals that the predicted protein sequence of *Daphnia *COX clusters with other invertebrates. There is no current evidence of an epoxygenase pathway in *Daphnia*; however, LOX products are most certainly synthesised in daphnids.

**Conclusion:**

We have outlined the structure of eicosanoid biosynthesis in *Daphnia*, a key genus in freshwater ecosystems. Improved knowledge of the function and synthesis of eicosanoids in *Daphnia *and other invertebrates could have important implications for several areas within ecology. This provisional overview of daphnid eicosanoid biosynthesis provides a guide on where to focus future research activities in this area.

## Background

**Figure 1:**
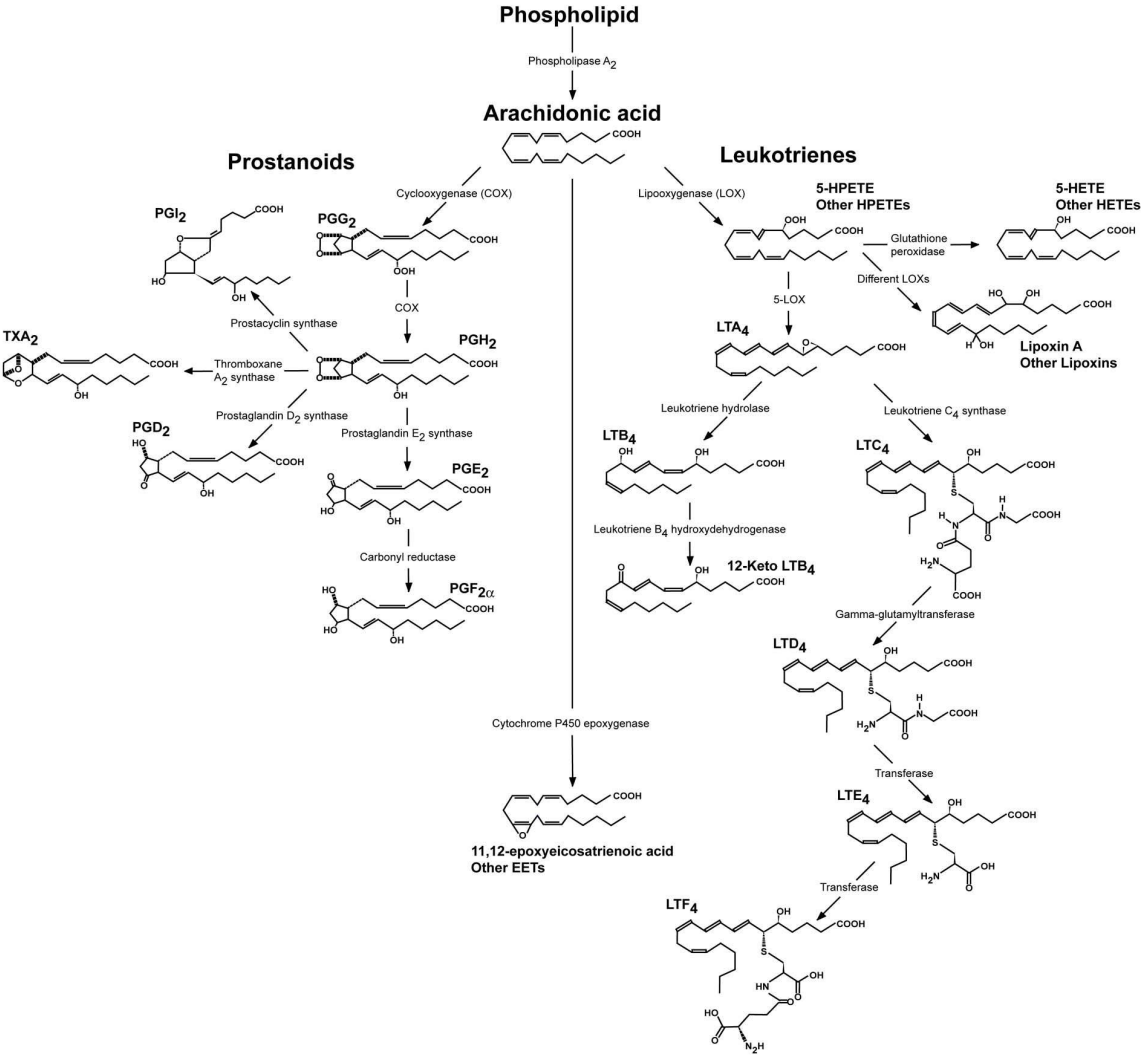
Overview of eicosanoid biosynthesis based on current knowledge from mammalian models. The three major pathways, cyclooxygenase (COX), lipoxygenase (LOX) and cytochrome P450 epoxygenase, are shown displaying major metabolites. Prostanoids cover prostaglandins (PG) and thromboxane (TX), while leukotrienes (LT) include LTs and lipoxins. PGD_2 _and PGE_2 _may be transformed into PGJ_2 _and PGA_2 _through either non-enzymatic rearrangement or dehydration, respectively. Abbreviations: EET, epoxyeicosatrienoic acids; HETE, hydroxyeicosatetraenoic acids; HPETE, hydroperoxyeicosatetraenoic acids. Diagram modified from Stanley [1] and KEGG [41].

Eicosanoids are cell signalling molecules derived from fatty acids acquired in the diet. Eicosanoid is a general term for all biologically active, oxygenated metabolites of three C20 polyunsaturated fatty acids; 20:3 n-6, 20:4 n-6 and 20:5 n-3. They have an important role in the regulation of essential functions such as reproduction and the immune system. All mammalian eicosanoids derive from a common precursor, arachidonic acid (AA), which is converted into eicosanoids with different functions through either the cyclooxygenase (COX) pathway (prostanoids: prostaglandins and thromboxane); the lipoxygenase (LOX) pathway (leukotrienes and lipoxins); or the cytochrome P450 epoxygenase pathway (epoxyeicosatrienoic acids) [1] (Fig. 1).

The COX enzyme exists in at least two isoforms in mammals, COX-1 and COX-2, which are inhibited by conventional non-steroidal anti-inflammatory drugs (NSAID) such as ibuprofen and aspirin, but only one COX isoform is generally present in invertebrates and lower vertebrates [2]. Mammalian COX-1 catalyses the generation of prostaglandins (PG) involved in many basic physiological functions such as regulation of blood pressure, gastric mucosal protection, maintenance of homeostasis, and reproductive and nervous system function; while PGs metabolised by COX-2 are involved in inflammation, ovulation and mitogenesis [3,4]. COX-1 is a constitutive enzyme (i.e. it is constantly expressed) found in the cell membranes of most mammalian tissues, while COX-2 is an inducible enzyme, not found in all cell types, that is located in nuclear membranes [1]. In the mammalian LOX pathway AA is converted by different LOXs into hydroperoxyeicosatetraenoic acids (HPETE) that may be further metabolised into hydroxyeicosatetraenoic acids (HETE) by glutathione peroxidase. Leukotrienes are synthesised from 5-HPETE by 5-LOX, while 8-LOX, 12-LOX and 15-LOX catalyse the production of different lipoxins from 8-HPETE, 12-HPETE and 15-HPETE (Fig. 1).

Research on eicosanoids has mainly been mammalian-driven and has lately been aimed at designing NSAIDs that are COX-2 selective due to the potential negative side-effects of COX-1 inhibition, which may affect the gastrointestinal tract, heart and kidneys [3]. Eicosanoids act at both the extracellular and intracellular level by interacting with distinct transmembrane G-protein coupled receptors (extra- and intracellular) and nuclear peroxisomal proliferator-activated receptors (PPAR) [5]. Activated G-proteins may, depending on cell type, stimulate second messengers such as cyclic adenosine monophosphate (cAMP) and/or intracellular calcium release [5]. PPAR are transcription factors which also have a role in ligand binding (eicosanoids), so directly influencing the expression of target genes involved in e.g. controlling prenatal and postnatal development [6]. These examples emphasise the biological significance of eicosanoids.

Less is known about eicosanoids in non-mammalian species; however, during the last three decades considerable evidence has been gathered concerning their synthesis and action. Eicosanoids have now been identified in almost every major metazoan phyla including some plants [1]. There is general consensus that eicosanoids act as autocrine or paracrine signallers (also referred to as local hormones) in both vertebrates and invertebrates, where they mainly function as important mediators in reproduction, the immune system and ion transport [1]. It is clear from a number of reports that eicosanoid generation is subject to inhibition by NSAIDs in a wide range of invertebrates [1,2]. Moreover, a COX derived mechanism similar to the mammalian biosynthesis of PGs has been proposed in the coral *Plexaura homomalla *[7]. There is also evidence of a LOX derived pathway being present in invertebrates based on the work of Ragab and colleagues [8] on the primitive wingless insect, the firebrat *Thermobia domestica*; although little is known about the structure of the pathway. Overall, invertebrate eicosanoid biosynthesis seems to have a simpler structure than its mammalian counterpart, as seen with the COX pathway [7], but also appears to be split into two instead of three pathways. There is currently no proof of an epoxygenase pathway being present in invertebrates [1].

There is little doubt that disruption of eicosanoid biosynthesis may upset many important physiological functions in both invertebrates and vertebrates, which can have serious consequences for both the individual and the population. Current evidence suggests that the main mode of action of the NSAID ibuprofen in *D. magna *relates to interruption of eicosanoid biosynthesis which reduces fecundity [9-12]. Eicosanoids may therefore play a pivotal role in daphnid reproduction. A wealth of synthetic and natural chemicals may affect invertebrate reproduction through endocrine disruption with one of the best known examples being imposex (masculinisation) of female molluscs caused by exposure to tributyl tin (TBT) [13]. It is therefore important to understand eicosanoid biosynthesis in *Daphnia*, and invertebrates in general, to fully recognize the potential mode of action of endocrine disrupters and how they may affect natural invertebrate populations.

Recently, the genome of *D. pulex *was fully sequenced [14], and action within the *Daphnia *Genomics Consortium has already been taken to start sequencing the *D. magna *genome [15,16]. In the meantime genes identified in *D. pulex *serve as a model for understanding eicosanoid biosynthesis, control and disruption in *Daphnia*. Here we present an overview of the putative eicosanoid biosynthesis in *Daphnia *based on annotation of genes from the *D. pulex *genome supported by recently published transcriptomic data (real-time quantitative PCR) of ibuprofen-stressed *D. magna *[12].

## Methods

Putative genes related to eicosanoid biosynthesis were identified on the *D. pulex *genome website [14] through using several bioinformatic search tools such as GO (Gene Ontology) [17], KEGG (Kyoto Encyclopedia of Genes and Genomes) [18] and matches against InterPro protein domains. Annotation of these genes were verified through BLAST searches performed against Swissprot protein and non-redundant protein sequence databases via the *D. pulex *genome website [14].

Invertebrate and vertebrate COX protein sequences were retrieved from GenBank [19] and Ensembl [20] for phylogenetic analysis. Since no arthropod COX protein sequences were available that could aid the phylogeny with respect to *D. pulex*, we searched the GenBank Expressed Sequence Tag (EST) division (est_others) using the BLAST algorithm (tblastn) to obtain additional arthropod COX sequences. Sequences that significantly resembled the sea squirt *Ciona intestinalis *COX amino acid sequence (E-value < 1e-20) were retrieved and included in the dataset. Six ESTs were obtained, representing two malacostracan species (Crustacea): Four ESTs were derived from *Homarus americanus *(accession numbers DV772953, DV774102, EH401871 and FD699680) and two were derived from *Petrolisthes cinctipes *(accession numbers FE773225, FE820815). After translating the nucleotide sequences to putative amino acids, it appeared that one *H. americanus *sequence (FD699680) did not overlap with the other three sequences. The three remaining sequences constituted two slightly deviating amino acid sequences; DV774102 differed from EH401871 and DV772953. The four *H. americanus *sequences were combined to one sequence with variable and missing positions specified as 'unknown'. Furthermore, the inferred amino acid sequences of the two *P. cinctipes *ESTs were similar, and *P. cinctipes *was thus included once in the dataset. Note that these additional crustacean entries were EST derived, single pass sequenced, and therefore not guaranteed to be free from sequencing errors.

The sequence COX dataset was aligned using ClustalW2 [21], manually edited after inspection and subsequently analysed using the Gblocks web-server [22] to pinpoint and remove unreliably aligned regions. This analysis allowed for gaps in the final alignment. ModelGenerator [23] was applied to obtain a model of sequence evolution (gamma distribution with four rate categories) using the Akaike Information Criterion. The predicted model, WAG+I+G, was specified in Phyml v2.4.4 [24] and a dendrogram was constructed using Maximum Likelihood (BIONJ [25] starting tree). Phylogenetic analysis used 100 bootstrap replicates. Finally, the obtained topology was visualised using TreeView [26].

## Results and Discussion

**Figure 2:**
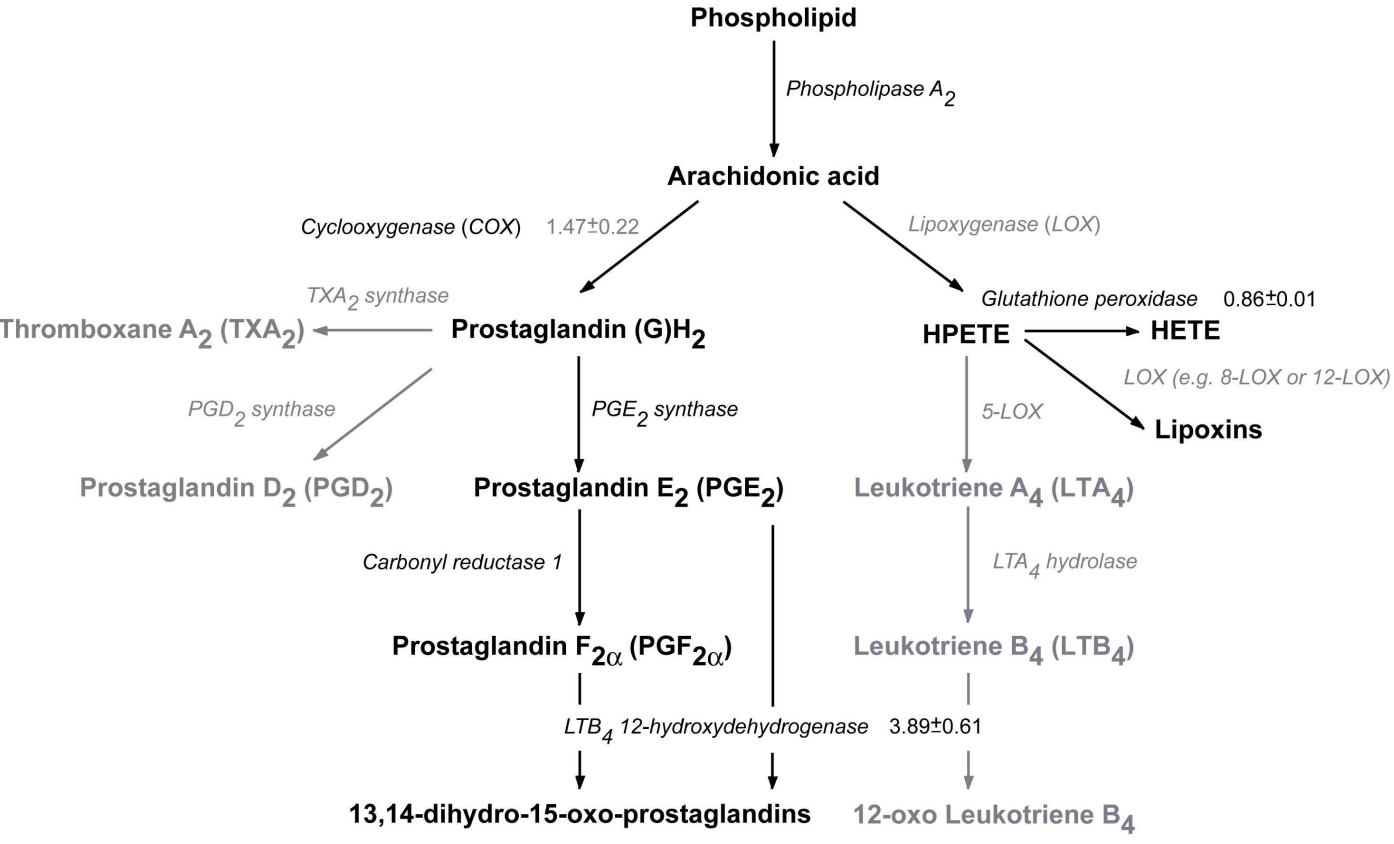
Putative eicosanoid biosynthesis pathway in *Daphnia *based on bioinformatic and transcriptomic evidence from *D. pulex *and *D. magna*. All the putative genes (names in italics) have been identified through different gene models in the *D. pulex *genome and are shown in black or grey font based on high (> 60%) or low (< 60%) similarity to proteins from other genomes (Table 1). Eicosanoids in grey font are less likely to be present in daphnids. Expression of ortholog genes in ibuprofen-stressed *D. magna *(24 h exposure to 20–80 mg l^-1^) was analysed using real-time quantitative PCR [12]. Fold change difference in gene expression (mean ± SE) is shown relative to controls (grey values are only weakly significant). The enzyme LTB4DH (encoded by *Ltb4dh*), that catabolises PGE_2_, PGF_2α _and LTB_4 _to become inactive eicosanoids, is also known as 15-oxo-prostaglandin 13-reductase. All the specified eicosanoids have been identified in other arthropod species [1,2], expect for the leukotrienes where only indirect evidence exits [37]. Abbreviations: HETE, hydroxyeicosatetraenoic; HPETE, hydroperoxyeicosatetraenoic acid. See text for further details.

Table 1 shows the putative genes related to eicosanoid biosynthesis that were identified through searching the *D. pulex *genome using several different bioinformatic tools (see Methods). Many of these genes had high similarity to their counterparts in higher organisms (Table 1). The bioinformatic evidence from *D. pulex *suggested that only the cyclooxygenase (COX) and lipoxygenase (LOX) pathways are present in *Daphnia *(Fig. 2) compared with the three pathways known from mammalian systems, i.e. the COX, LOX and epoxygenase pathways. This agrees with earlier findings as no epoxygenase pathway has been identified in invertebrates to date [1].

**Table 1:** Putative *Daphnia pulex *genes associated with eicosanoid biosynthesis

**Putative gene^a^**	**Protein ID^b^**	**Protein size ** **(AA)**	**Best match ** **(species)^c^**	**Accession** **number**	**Similarity ** **(%)**	**E-value**
Phospholipase A2	DAPPUDRAFT_5959	129	*Apis mellifera*	XP_624621	76	0.0
Cyclooxygenase	DAPPUDRAFT_313427	689	***Sus scrofa***	NP_999486	62	0.0
Prostaglandin D2 synthase A	DAPPUDRAFT_307787	211	*Tribolium castaneum*	XP_970647	49	2.5E-35
Prostaglandin D2 synthase B	DAPPUDRAFT_316534	228	*T. castaneum*	XP_967406	54	2.3E-32
Prostaglandin E2 synthase	DAPPUDRAFT_56335	283	*Aedes aegypti*	EAT39424	61	0.0
Carbonyl reductase 1 (PG 9-ketoreductase)	DAPPUDRAFT_310758	257	***Xenopus laevis***	Q6DJN9	76	0.0
*Thromboxane A*	DAPPUDRAFT_96715	463	*Homarus americanus*	AAC28351	49	0.0
*Thromboxane B*	DAPPUDRAFT_328913	501	*H. americanus*	AAC28351	59	0.0
Lipoxygenase 1	DAPPUDRAFT_311736	562	*T. castaneum*	XP_969219	48	0.0
Lipoxygenase 2	DAPPUDRAFT_95367	433	***Bos taurus***	XP_593842	33	2.1E-43
Glutathione peroxidase	DAPPUDRAFT_337058	219	*Ixodes scapularis*	AAY66814	67	0.0
*Leukotriene A4 hydrolase*	DAPPUDRAFT_313156	619	***X. tropicalis***	NP_001006898	67	0.0
Leukotriene B4 12-hydroxydehydrogenase	DAPPUDRAFT_311788	340	*A. aegypti*	EAT35376	65	0.0
Prostanoid receptor EP4 isoform A	DAPPUDRAFT_58618	166	***Danio rerio***	LOC560057	59	3.6E-38
Prostanoid receptor EP4 isoform B	DAPPUDRAFT_58558	121	***Tetraodon nigroviridis***	CAF96390	60	4.2E-30

^a^Gene names displayed in italics are unlikely to be involved in daphnid eicosanoid biosynthesis (see Fig. 2 and text for further information); ^b^*D. pulex *protein ID is linked to a GenBank [19] accession no.; ^c^Vertebrate species are signified by bold font, while invertebrate matches are denoted by normal font.

**Figure 3:**
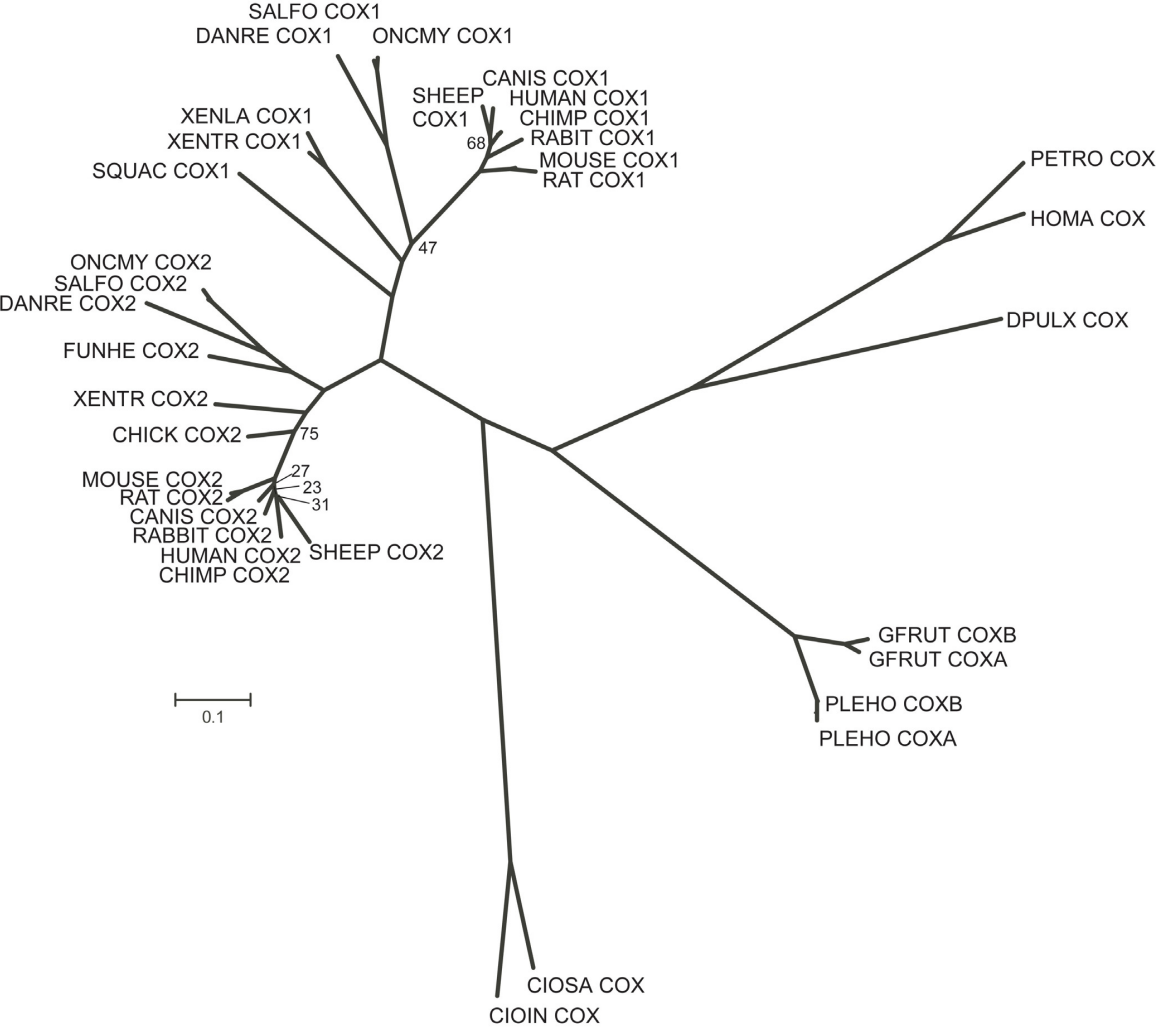
Phylogenetic tree of cyclooxygenase (COX) based on protein sequences from a diverse range of organisms constructed using Maximum Likelihood. All bootstrap values above 80 have been removed. Scale: 0.1 substitutions per site. Sequences can be retrieved from GenBank [19] or Ensembl [20], while the *Daphnia pulex *COX was derived from a predicted protein sequence based on the best gene model available on the *D. pulex *genome portal [14]. Note that some sequences are based on EST information (see Methods). Abbreviations (accession no. in brackets with COX1 shown first when there are two no.): CANIS, *Canis familiaris *(NP_001003023; NP_001003354); CHICK, *Gallus gallus *(P27607); CIOIN, *Ciona intestinalis *(ENSCINT00000012734); CIOSA, *C. savignyi *(ENSCSAVT00000000782); DANRE, *Danio rerio *(Q8JH44; Q6P4V3); DPULX, *Daphnia pulex *(NCBI_GNO_0900053); FUNHE, *Fundulus heteroclitus *(Q6QNF2); GEFRU, *Gersemia fruticosa *(Q9GPF4; Q6S375); HOMA, *Homarus americanus *(EST: DV772953, DV774102, EH401871 and FD699680); HUMAN, *Homo sapiens *(P23219; P35354); MOUSE, *Mus musculus *(Q543T1; Q05769); ONCMY, *Oncorhynchus mykiss *(Q9DEQ0; Q9W715); PANTR, *Pan troglodytes *(ENSPTRT00000042391; ENSPTRT00000003246); PETRO, *Petrolisthes cinctipes *(EST: FE773225, FE820815); PLEHO, *Plexaura homomalla *(Q962I8; Q5IX63); RABIT, *Oryctolagus cuniculus *(O97554; O02768); RAT, *Rattus norvegicus *(Q63921; Q63124); SALFO, *Salvelinus fontinalis *(Q9PTN3; Q9PW89); SHEEP, *Ovis aries *(P05979; P79208); SQUAC, *Squalus acanthias *(Q8UVQ3); XENLA, *Xenopus laevis *(A0A9J3); XENTR, *X. tropicalis *(ENSXETT00000035660; Q501R2).

Both the COX and LOX pathways in *Daphnia *appeared to have a simpler structure than their mammalian counterparts (for comparison, see Figs. 1 and 2). For instance, there was no bioinformatic evidence of prostacyclin synthase, which converts PGG_2 _into PGI_2_, in the daphnid COX pathway. The gene encoding this enzyme was likewise not identified in the genome of the urochordate *C. intestinalis *[2]. Additionally, there was only bioinformatic evidence of one gene encoding COX in the *D. pulex *genome. A phylogenetic comparison of the predicted *D. pulex *COX with other protein sequences revealed that daphnid COX clusters with the invertebrates being most closely related to other crustaceans. The COX phylogeny likewise showed that COX-1 and COX-2 comprise two distinct clades amongst the vertebrates (Fig. 3). Generally, it is understood that invertebrates and lower vertebrates only have one non-specific type of COX [2], but recently two COX isoforms have been identified in the corals *Plexaura homomalla *[7] and *Gersemia fruticosa *[27]. Rowley et al. [2] suggest that the COX genes found in corals are an early version that predates the (supposed) vertebrate duplication into the typical constitutive COX-1 and inducible COX-2 isozymes found in vertebrates. Only one copy of the COX gene has been identified in the *C. intestinalis *genome [2] which, as a member of the Phylum Chordata, shares a more recent common ancestor with the vertebrates. This was supported by our phylogenetic analysis (Fig. 3) suggesting that a duplication of the COX gene occurred in the Chordata. Rowley and colleagues further speculate that the evolution of COX-1 and COX-2 probably predates the emergence of bony fish some 350 million years ago [2]. This is supported by the fact that only one constitutively expressed COX isoform has been identified in the shark *Squalus acanthias *(Spiny dogfish) [2]. In our phylogenetic analysis *S. acanthias *COX clustered with the vertebrate COX-1 clade.

Like the putative *Daphnia *COX pathway, the structure of the daphnid LOX pathway also appeared to be more simple than its mammalian counterpart, which confirms previous findings [1]. There was gene sequence evidence that indicated the presence of three leukotrienes (LT) in *Daphnia*: LTA_4_, LTB_4 _and 12-oxo LTB_4 _(Table 1). This is, however, doubtful since no LTs, nor the precursor 5-HPETE, have been identified in invertebrates and lower vertebrates to date [28]. It is possible that the putative LTA_4 _hydrolase (LTA4H), which is a bi-functional enzyme in mammals, having both LTA_4 _hydrolase and aminopeptidase activity, only has aminopeptidase activity in daphnids as has been reported in *Caenorhabditis elegans *[29]. However, recent evidence shows that bi-functionality of LTA4H is only six point mutations away in yeast compared to the mammalian enzyme [30]. There is reason to believe that daphnid LTA4H may be bi-functional, and thus able to convert LTA_4 _into LTB_4_; a hypothesis that we will test in future experiments. Furthermore, transcriptomic evidence from *D. magna *shows that the expression of *leukotriene B4 12-hydroxydehydrogenase *(*Ltb4dh*) increases with increasing concentration of the eicosanoid-inhibiting drug ibuprofen [11]. This does not prove that LTs are present in daphnids, but yet again we cannot rule out the possibility. The enzyme (LTB4DH) encoded by *Ltb4dh *is also bi-functional in mammals regulating eicosanoids by rapidly degrading different E and F series PGs and LTB_4 _[31] (see below). The former function of LTB4DH (PG catabolism) appears to be the most likely in daphnids until solid proof exits about the presence of LTs (Fig. 2).

The presence of lipoxins is, however, more likely, and bioinformatic information from *D. pulex *suggests that at least two LOX enzymes are present (Table 1). LOX enzymes have been found in all organisms studied, from bacteria to man. The two putative *D. pulex *LOXs are both composed by two domains; an N-terminal lipase domain belonging to the InterPro protein family 734 (IPR000734) and a C-terminal LOX LH2 domain (IPR001024). This resembles mammalian LOX enzymes that are also comprised of two domains; a regulatory N-terminal domain that is similar to mammalian lipases and a catalytic LOX domain (C-terminal) [32]. LOX LH2 is the only LOX related domain that has been identified in the *D. pulex *genome. Other known LOX domains include e.g. mammalian LOX (IPR001885) and LOX, C-terminal (IPR013819). There are 13 *D. pulex *gene models that contain the LOX LH2 domain [14], but only two genes contain both an N-terminal domain similar to mammalian lipases (IPR000724) and a C-terminal LOX domain (Table 1). Further investigations are needed to specify what type of LOX enzyme these two *Daphnia *sequences represent prior to analysing their phylogenetic relationship. However, it is likely that they could be 8-LOX and 12-LOX, which synthesise different lipoxins from 8-HPETE and 12-HPETE, as these LOXs have been identified in a range of invertebrate species [8,33,34].

Two enzymes, 15-hydroxyprostaglandin dehydrogenase (PGDH) and LTB4DH are known to irreversibly inactivate bioactive eicosanoids in mammals. Both enzymes are key in regulating the hormonal-like action of eicosanoids by rapidly degrading PGE_2_, PGF_2α_, and LTB_4_, as overproduction of these potent mediators may have serious physiological effects such as initiating inflammation [31]. It appeared that LTB4DH fulfils this regulatory function single-handedly in daphnids, as there was no indication of PGDH being present (Table 1, Fig. 2).

The bioinformatic and transcriptomic evidence from *D. pulex *and *D. magna *(Fig. 2) suggests that PGs (e.g. PGH_2_, PGE_2 _and PGF_2α_), lipoxins and possibly LTs could be present in daphnids. Low similarity of TXA_2 _synthase and PGD_2 _synthase to ortholog proteins from other genomes (Fig. 2) render the presence of PGD_2 _and TXA_2 _to be less certain in daphnids, although this could merely be due to daphnids having more divergent versions of these proteins. Nevertheless, it seems most likely that daphnids do not produce TXA_2 _since the gene encoding TXA_2 _synthase has not been identified in the genome of *C. intestinalis *[2]. The LOX encoding genes identified in *D. pulex *were also slightly doubtful due to the same reasons, but it would be more probable that these enzymes are present in *Daphnia *as both 8-LOX and 12-LOX derived lipoxins are common in invertebrates [8,33,34]. PGA_2 _may also be present in daphnids as it is non-enzymatically rearranged from PGE_2 _and has been detected in several arthropods [1]; but until verified by mass spectrometry or the like it remains speculative what eicosanoids are present in *Daphnia*. Moreover, the annotation of genes from the daphnid eicosanoid biosynthesis (and other daphnid genes for that matter) should improve as more invertebrate genomes become sequenced and annotated.

The possible roles of eicosanoids in daphnids have already been suggested from several invertebrate studies, including *D. magna *[12], where both prostanoids and lipoxygenase products appear to be important agents in oogenesis (especially vitellogenesis) and embryogenesis [35-38]. For instance, PGE_2 _is known to initiate egg-laying behaviour in several insect species (e.g. orthopterans), where it seems to regulate muscle contractions in the ovarian musculature [39]. Furthermore, many of the above-mentioned eicosanoids have likewise been identified as important mediators in arthropod immune systems (both COX and LOX products) [40] and ion transport physiology (mainly PGE_2 _and PGF_2α_) [1]. Until more integrated phenotypic and genomic evidence exists it is difficult to infer an exact role for eicosanoids in daphnids, as they may be involved in several processes and act in different tissues. Nevertheless, it is almost certain that eicosanoids play vital roles in the functioning of processes key to daphnid reproduction and survival [9,10,12]. Finally, bioinformatic evidence from the *D. pulex *genome also revealed that two prostanoid G-protein coupled receptors may be present, thus further supporting the evidence that eicosanoids are bioactive agents in daphnids (Table 1).

## Conclusion

Eicosanoids are key molecules, involved in the function of fundamentally important biological systems. A better understanding of their biochemistry and genetic control in invertebrates will help to improve our understanding of their significance in these organisms. Here we have outlined a putative structure of eicosanoid biosynthesis in *Daphnia*, a key macroinvertebrate in freshwater ecosystems. It would seem, from transcriptomic and phenotypic evidence, that eicosanoids play a pivotal role in daphnid reproduction [12]; but their importance in other physiological functions such as the immune system remains to be investigated. Improved knowledge of the function and synthesis of eicosanoids in *Daphnia *and other invertebrates could have very important implications for several areas within ecology including ecological risk assessment. This provisional overview of daphnid eicosanoid biosynthesis provides a guide on where to focus future research activities in this area.

## Competing interests

The authors declare that they have no competing interests.

## Authors' contributions

LHH and MJTNT performed the bioinformatic and phylogenetic analyses. LHH drafted the manuscript. All authors contributed intellectually to the manuscript and approved the final version.
